# Comparing the Usability of the Web-Based 24-h Dietary Recall R24W and ASA24-Canada-2018 among French-Speaking Adults from Québec

**DOI:** 10.3390/nu14214543

**Published:** 2022-10-28

**Authors:** Catherine Laramée, Simone Lemieux, Julie Robitaille, Benoît Lamarche

**Affiliations:** Centre Nutrition, Santé et Société (NUTRISS), Institut sur la Nutrition et les Aliments Fonctionnels (INAF), Université Laval, Québec, QC G1V 0A6, Canada

**Keywords:** dietary recall, dietary assessment, online, usability, user experience, ASA24, R24W

## Abstract

Automated, self-administered, Web-based 24-h dietary recall tools are increasingly available for nutrition research in different settings, particularly in epidemiological studies and national surveys because of their practicality and efficiency. However, the usability of different 24-h dietary recall tools must be assessed and compared for use in specific populations as it is a major driver of the response rate and retention of participants. The primary aim of this study was to compare the usability of two validated, self-administered, web-based 24-h dietary recall tools available for the Canadian population: the R24W and the 2018 Canadian version of the ASA24. The R24W was developed in French for primary use in the province of Québec, Canada while the ASA24 was developed in English for primary use in the USA and recently adapted and translated for use in French-speaking Canadian adults. Whether the R24W and the ASA24-Canada-2018 yield similar nutritional data was also tested. In this randomized crossover study, 48 women and 20 men (mean age of 35 ± 14 years; range: 19–79 years) recruited in the province of Quebec completed the R24W and the ASA24-Canada-2018 in French twice on each occasion. Participants also completed the System Usability Scale (SUS), a reliable and valid scale giving a global view of subjective assessments of usability. Mean SUS score as well as mean dietary intakes of energy, nutrients and food groups generated by each tool were compared using mixed model analyses for repeated measures. On a scale of 0 to 100, the mean SUS scores (±SD) for the R24W and the ASA24-Canada-2018 were 81 ± 2 and 58 ± 2, respectively (*p* < 0.0001). 84% of participants stated that they would prefer to use the R24W if they were invited to complete additional 24-h dietary recalls. No significant difference was found between the R24W and the ASA24-Canada-2018 for the intake of energy, proteins, lipids, saturated fatty acids, carbohydrates, fibers, sodium and vegetables and fruits. In sum, while the R24W and the ASA24-Canada-2018 generate comparable self-reported dietary intake data, the R24W showed a better usability than the ASA24-Canada-2018 in a sample of French-speaking adults from the province of Quebec.

## 1. Introduction

Accurate assessment of dietary intake and representativeness of the sample population are key to understanding the relationship between diet and health outcomes and to evaluate the impact of nutrition-focused public health policies. Validation studies using biomarkers have demonstrated that repeated 24-h dietary recalls provide high quality data with minimal systematic bias [1,2,3]. However, traditional 24-h dietary recalls are expensive as they rely on trained interviewers for data collection and coding. For those reasons, researchers have developed automated, self-administered, web-based dietary recall tools that are increasingly available for nutrition research, particularly for epidemiological studies and national surveys [4,5,6,7]. 

The “Rappel de 24 h Web” (R24W) [8] and The Automated Self-Administered 24-h (ASA24) [9] are two automated, self-administered, web-based dietary recall tools available in Canada in French and in English. The R24W was developed in French at Université Laval in Québec City and released in 2016 whereas the ASA24 was developed in English at the US National Cancer Institute and adapted for use in Canada by Health Canada’s Food Directorate in 2014 and then updated in 2016 and 2018. The ASA24 was also translated in French in 2016. The R24W and the ASA24-Canada-2018 have a different but diversified food list representative of the Canadian population’s food habits, are both linked to the Canadian Nutrient File food composition database (v2015) and use food portion pictures to help respondents estimate portions sizes (Table 1). The R24W refers to 2669 foods from which 148 nutrients can be automatically extracted. The ASA24-Canada-2018 refers to 4794 foods from which 65 nutrients can be automatically extracted. The R24W has an optional supplement intake module that collects qualitative information on the type of supplements consumed while the supplement intake module of the ASA24-Canada-2018 collects both qualitative and quantitative information about supplements types and doses. The main methodological difference between the R24W and the different versions of the ASA24 including the ASA24-Canada-2018 relates to the order of the Automated Multiple-Pass Method [10] steps that respondents must follow to record dietary assessment (Table 1). While the R24W asks for details after each selected food [8], the ASA24 asks for details once all the foods of a given meal have been selected [11]. Both tools have been validated using various methods and populations. For example, in two fully controlled feeding studies, nearly 90% and 80% of the food items provided were reported using the R24W and the ASA24, respectively [12,13]. The portion sizes reported did not differ significantly from the portion sizes offered and it did not have a significant impact on energy intake estimate (−13.9 kcal *p* = 0.83 for R24W and 125 kcal *p* = 0.34 for ASA24) [12,13]. 

However, usability of the R24W and the ASA24-Canada-2018 have not been formally tested and compared in French-speaking populations. This is important because usability may be a major driver of the response rate and retention of participants in a study, hence influencing the quality of the data being collected.

According to the International Organization for Standardization (ISO 9241-11), usability is the extent to which a system can be used by specified users to achieve specified goals with effectiveness (i.e., accuracy and completeness), efficiency, and satisfaction in a specified context of use [14]. Efficiency relates to the resources used (e.g., time and human effort) in relation to the results achieved while satisfaction describes the extent to which the user experience meets the user’s needs and expectations. In other words, usability is the overall experience and ease of use of the system by users. Self-administered web-based dietary assessment tools have two types of users: the respondents who report dietary intakes and the researchers who manage the study logistics and analyze the data. Usability can be assessed by quantitative-retrospective methods (e.g., questionnaires), quantitative-real-time methods (e.g., task completion), qualitative-retrospective methods (e.g., interview or focus groups) or qualitative-real-time methods (e.g., “Think-Aloud” protocol) [15]. 

The primary aim of this study was to compare the usability from the respondent’s perspective of the R24W and the ASA24-Canada-2018, which is the latest Canadian version, in a sample of French-speaking adults when used on a computer or a tablet in a real-life setting. We also took the opportunity to test for the first time whether the two web-based 24-h recall tools generate comparable dietary intake data. We hypothesized that the usability score of the R24W is higher than the ASA24-Canada-2018 because the former was developed in Québec specifically for the French-speaking population. We also hypothesized that both tools generate comparable dietary intake data.

## 2. Materials and Methods

### 2.1. Participants

Participants were recruited between November 2019 and October 2021 through Facebook adds and email lists of employees and students available at Université Laval. Men and women of 18 years of age and over with access to Internet and to a computer or a tablet were eligible. Participants also had to have an active email address and be able to complete questionnaires in French. Being a dietitian or a student in nutrition as well as having already used the R24W or the ASA24 in the past were exclusion criteria because we wanted to test the initial performance with the tools (initial learnability) [16]. 

### 2.2. Study Design

This study was undertaken using a randomized crossover study design stratified by sex. As the R24W and the ASA24-Canada-2018 are typically administered in a research context along with other questionnaires, participants were asked to complete questionnaires assessing potential moderating variables such as health literacy and socio-demographic characteristics at baseline. Participants had one week to complete the baseline questionnaires. During the subsequent two-weeks, participants were asked to complete two non-consecutive dietary recalls on unannounced, randomly selected weekdays or weekend days using the French version of either the R24W or ASA24-Canada-2018 according to the sequence assigned. Over the next two weeks, participants were asked to complete two other dietary recalls using the second recall tool. Up to three reminders were sent when recalls were not completed by the initial scheduled date. This approach reflects the settings of prospective studies in which repeated administrations of dietary recalls on non-consecutive days is recommended to examine the association between diet as an independent variable and a health outcome as a dependent variable [17]. Participants were asked to complete the usability questionnaire after having completed each set of recalls before using the second tool. Informed consent was obtained from all subjects involved in the study. The study was conducted according to the guidelines of the Declaration of Helsinki, and approved by the Ethics Committee of Université Laval (2019-226 R-2/23 September 2021).

### 2.3. Dietary Recalls Tools

As described in the introduction, the R24W and the ASA24-Canada-2018 share a lot of similarities but also have important difference (Table 1). In the context of the present study, both tools were configured to be as similar as possible in terms of modules integrated, intake time frame, etc. (see Table S1). Participants were asked to complete the dietary recalls on a computer or a tablet because the R24W was not adapted for smartphone at the beginning of this study. No support was provided to complete the recalls.

### 2.4. Outcome Measurements

#### 2.4.1. Usability

Participants were invited to complete a free translation of the System Usability Scale twice, once after using the R24W and once after using the ASA24-Canada-2018. The SUS is a reliable and valid scale giving a global view of subjective assessments of usability [18,19]. It is a 10-item scale using a 5-point Likert scale ranging from strongly disagree to strongly agree. Examples of items include “I found the system unnecessarily complex”, “I thought the system was easy to use” and “I found the various functions in this system were well integrated”. SUS yields a single score from 0 to 100 representing a composite measure of the overall usability of a system and allow to ordinally compare two or more systems. A higher score indicates higher usability. 

An open-ended question on the most and least appreciated aspects of the tool was added at the end of the SUS questionnaire to further document potential differences between the SUS score of the R24W and the ASA24-Canada-2018. At the end of the study, participants were also asked: “If I had to complete other 24-h dietary recalls, I would prefer to use…”. The answer choices were “The R24W” or “The ASA24”. Finally, completion times (total session duration) were extracted as a measure of efficiency. 

#### 2.4.2. Dietary Intakes

Mean dietary intakes of energy, macronutrients and other key nutrients (saturated fat, fibers and sodium) as well as the average number of the 2007 Canada’s Food Guide servings were automatically calculated by the R24W and the ASA24-Canada-2018. 

#### 2.4.3. Health Literacy

At baseline, participants were invited to complete the Canadian adaptation of the Newest Vital Sign (NVS) to assess health literacy [20]. The NVS is a validated and reliable tool comprising 6 questions that assesses an individual’s ability to find and understand both text and numerical information on the nutrition label of an ice-cream container [20,21]. Based on the number of correct answers, the respondent is categorized as having a high likelihood of low health literacy (score 0–1), possible low health literacy (score 2–3) or adequate health literacy (score 4–6).

#### 2.4.4. Socio-Demographic Characteristics

The baseline socio-demographic questionnaire included questions on age, sex, height, weight, ethnicity as well as annual gross household income and education level as surrogates of socioeconomic status.

### 2.5. Sample Size Estimation

Based on previous studies [22,23] and considering a dropout rate of 10%, sample size calculations indicate that a total of 74 participants would yield a power of 80% to detect a minimal difference of 5 points on a scale of 100 (*p* < 0.05) in SUS score (the study primary outcome) between the R24W and the ASA24-Canada-2018. We considered a 5% difference in usability score to be of significance. 

### 2.6. Statistical Analyses

The SUS score was calculated in accordance with Brooke [18]. Mean SUS score and mean dietary intakes generated by each tool were compared using mixed models for repeated measures. The main fixed effect was the recall tool (R24W vs. ASA24-Canada-2018) compared in paired analyses with subject as a random effect. While the study was not powered to investigate interactions, the moderating effects of sex and age were explored using proper interaction terms. Characteristics of participants and dropouts as well as the preferred recall tools (expressed in percent) were compared using chi-square test. The median completion times for each tool were compared using Wilcoxon test. Statistical analyses were done in SAS^®^ (SAS OnDemand for Academics). An inductive content analysis of the open-ended question about the most and least appreciated aspects of the tools was also conducted by one coder by grouping similar ideas in Excel.

## 3. Results

### 3.1. Participant Characteristics

**Figure 1:**
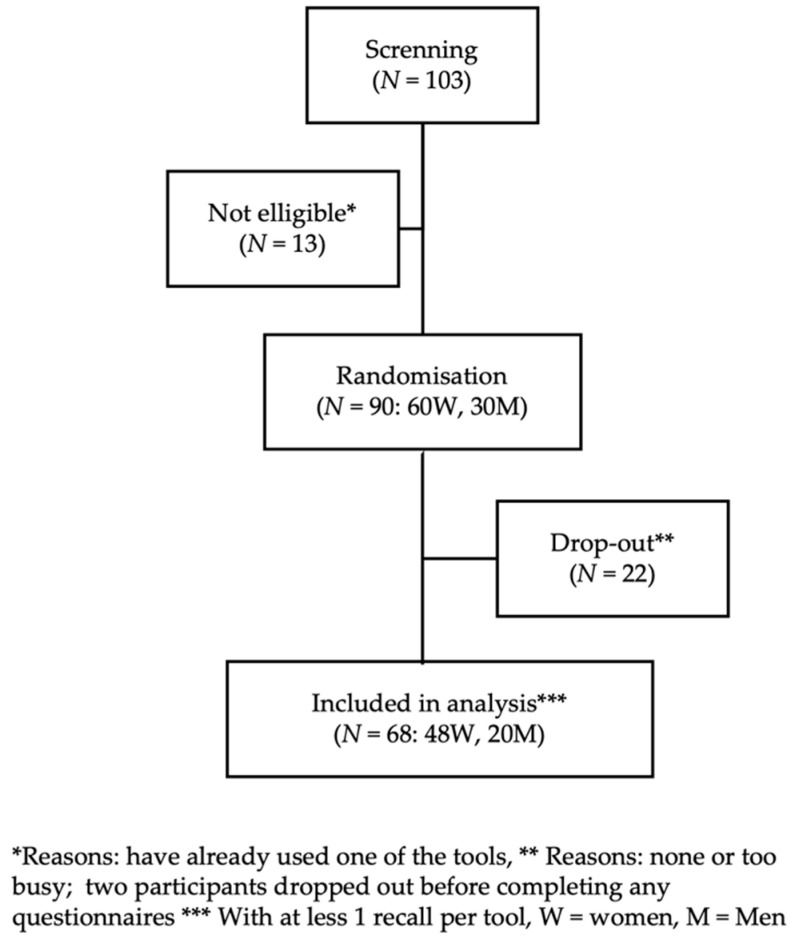
Study flow-chart.

Although the strategy was to recruit the same proportion of men and women, a total of 60 women and 30 men were recruited and randomized by sex to ensure an equal number of men and women within each treatment sequence. The drop-out rate was 24% (Figure 1). As a result, a total of 48 women and 20 men (mean age of 35 ± 14 years; range 19–79 years) were included in the analyses (Table 2). A little over half of the participants reported having a University education and the health literacy was considered “adequate” for all participants. Age, sex, education and income were similar between participants and dropouts. 

### 3.2. Usability

The mean (±SD) SUS scores for the R24W and ASA24-Canada-2018 on a scale of 0 to 100 were 81 ± 2 and 58 ± 2, respectively (*p* < 0.0001, Table 3). 84% of participants indicated that they would prefer to use the R24W rather than the ASA24-Canada-2018 if they were invited to complete additional dietary recalls. The median (interquartile range) completion time for the R24W and ASA24-Canada-2018 were 14 min (9–19) and 25 min (16–34), respectively (*p* < 0.001). 

Photos of the portions, variety of foods and reminders about frequently forgotten foods and potential additions (e.g., sauces and toppings) were among the most appreciated features of both 24-h recall tools while absence of certain foods was among the least appreciated aspects (Table 4). Visual appearance was among the most appreciated features of the ASA24-Canada-2018 and among the least appreciated feature of the R24W. Not having the possibility to create one’s own recipe or to add a food manually was another feature of the R24W that was less appreciated. The number of steps, details and questions, having to describe portions after listing all foods and the difficulty to find foods with the search function were the least appreciated features of the ASA24-Canada-2018. Simplicity and ease of use were the most appreciated features of the R24W. 

### 3.3. Dietary Intakes

The mean number of food items reported were similar between the R24W and the ASA24-Canada-2018 (18.2 ± 6.4 and 18.6 ± 7.6, *p* = 0.63). As shown in Table 5, no significant difference was found between the R24W and ASA24 for the self-reported intake of energy, macronutrients, proteins, lipids, saturated fatty acids, carbohydrates, fibers, sodium and of Canada’s 2007 Food groups.

## 4. Discussion

The present study revealed that the R24W obtains a higher usability score than the ASA24-Canada-2018 in a sample of French-speaking adults from the province of Quebec. The difference in SUS score appears to be due to greater self-reported ease of use and lower completion time for the R24W compared to the ASA24-Canada-2018. Both dietary recall tools yielded comparable self-reported dietary intake data.

Usability was assessed using the SUS administered after experiencing each recall tools. The SUS is one of the most common questionnaires used to evaluate a variety of softwares including in the field of nutrition [7,15]. The SUS score of the R24W was 23 points higher than the SUS score of the ASA24-Canada-2018. According to Bangor et al., SUS scores at least equal to 70 can be considered “good” which was the case for the R24W but not for the ASA24-Canada-2018 in this population [24]. The fact that the R24W was developed in Quebec specifically for the French speaking adult population probably contributes to its good usability. The SUS has also already been used to assess the usability of the 2014 Canadian version of the ASA24 in a subset of participants aged 48 to 67 years from the Canadian Longitudinal Study on Aging cohort. Very similar to data from the present study, the mean SUS score was 57 and half of the participants reported finding the ASA24-Canada-2014 unnecessarily complex [22]. The range of the scores obtained with the R24W and the ASA24-Canada-2018 are also consistent with those obtained for other 24-h recall tools available in other countries. For example, myfood24 developed in United Kingdom had a SUS score of 55 in older adults [25], of 59 in a clinical population [26], of 71 in women with gestational diabetes [23] and of 74 in an adolescent population [27]. 

The analysis of the open-ended question on the most and least appreciated features of each recall tools identifies potential areas to improve each dietary assessment tool when targeting a French-speaking population. For example, improving the visual appearance of the R24W and adding a functionality to create a recipe or to add food manually would most likely further increase its usability. The food search function of the ASA24-Canada-2018 was considered suboptimal, which is consistent with a usability study of the 2016 Canadian version of the ASA24 among low-income adults in British Columbia, Canada [28]. Of note, the 2020 US version of the ASA24 has improved its food search function [29], and this may contribute to higher usability scores. Even though the variety of food choice available was identified among the most appreciated features for each of the tools, the absence of certain foods was also identified as one of the least appreciated features of both tools. This suggests that broadening the food database would likely increase usability for both the R24W and the ASA24-Canada-2018. According to a recent literature review, the ability to easily identify the correct food is one of the most common issues raised in web-based 24-h recall tools usability studies [7]. In the next few years, the integration of more branded products into these tools should be considered to facilitate the identification of the exact food consumed while providing many descriptors (e.g., country of origin and presence of nutrition or health claim) with less data management. However, the challenges remain to link each branded food to full nutritional composition, to maintain an effective search function with expanded database without increasing the time required to complete the recall and to keep the food database up to date. 

The present study revealed no difference between the R24W and ASA24-Canada-2018 for the self-reported intake of energy, proteins, lipids, saturated fatty acids, carbohydrates, fibers, sodium as well as of several food groups. This is not surprising considering that both are validated and were developed with similar objectives and structures. Specifically, both 24-h recall tools use the same nutritional database and use food portions pictures to help in estimating portions size. Participants also reported a similar mean number of food items with the R24W and the ASA24-Canada-2018. Despite that, the median completion time was 11 min shorter with the R24W than with the ASA24-Canada-2018 (14 vs. 25 min, respectively). The median completion of the ASA24-Canada-2018 is essentially similar to the median completion time of 24 min reported on the ASA24 Website [30]. The shorter median completion time with the R24W compared to the ASA24-Canada-2018 probably contributed to its higher usability score.

This study’s strengths include the randomized cross-over study design, the real-life setting and the use of a validated usability questionnaire. Participants had relatively high socioeconomic and health literacy status, which limits the generalization of the results. Despite the advantages of automating dietary recalls, eliminating interviewer contact may increase the difficulty of collecting dietary intake data from the respondents’ perspective, particularly in subgroups with lower socioeconomic status [28] and with older people [22,25]. The study was conducted on computer or tablet using the French versions of the recall tools, which also limits generalizability to the cellphone and to the English versions of these tools. Finally, usability from the researcher’s perspective was not formally tested. 

## 5. Conclusions

While the R24W and the ASA24-Canada-2018 generate comparable self-reported dietary intake data for the nutrients compared, the R24W showed a better usability than the ASA24-Canada-2018 in a sample of French-speaking adults from the province of Quebec. Further research is needed to determine the usability of the R24W in other Canadian provinces, in English-speaking populations, in older adults and in lower socioeconomic populations.

## Figures and Tables

**Table 1:** Comparison of the Characteristics of the Respondents Website of the R24W and the ASA24-Canada-2018.

	R24W	ASA24-Canada-2018
Developer	Université Laval, Quebec, Canada	US National Cancer Institute, adapted by Health Canada’s Food Directorate
App languages	Developed in French and translated in English	Developed in English and translated in French
Tutorial	Mandatory video	Written instructions
Steps	Modified version of the Automated Multiple-Pass Method (AMPM) Enter meal or snack and context Enter a food or drink Select the food portion size Select the food additions or toppings Return to step 2 Review frequently forgotten food for the meal or snack Return to step 1 Final Review Water intake (optional) Usual Intake question (optional) Adherence to dietary regimen (optional) Supplements and Natural health product intakes (optional; qualitative information only)	Modified version of the AMPM Meal-based Quick List: Enter one by one all meals and snacks, their context and all foods, drinks, and dietary supplements eaten for each meals or snacks Meal gap review Detail pass: Question about Portion size, Preparation, Additions, Source (optional) of all the foods Final review Review frequently forgotten food Last chance Usual Intake question Supplement intake Module (optional) Sleep Module (optional)
Food identification	Search function (including synonyms, brand names and common misspelling) or; Browse food categories (hierarchical tree)	Search function (including synonyms, brand names and common misspelling) + filtering foods by categories
Food list	2669 food items including 687 recipes; Generic foods typically consumed in the province of Quebec	4794 food items; Personal recipe Fast food restaurant items
Integrated food composition database	Canadian Nutrient File from Health Canada (CNF, 2015) Homemade recipe database U.S Department of Agriculture (USDA) database when the food was not available in CNF	CNF, 2015 Canadian recipe database Food and Nutrient Database for Dietary Studies (FNDDS) database when the food was not available in CNF National Health and Nutrition Examination Survey (NHANES) Dietary Supplement Database 2007–2008 Licensed Natural Health Products Database
Portions sizes	Description in unit, volume or weight (Metric system) 4 images for most food	Description in unit, volume or weight (Imperial system) Participants can choose how they want to report quantity (eg. Whole fruit, cup, slices) 8 images for most food
Data Reports	Quantity of food and drink consumed Intake of 148 nutrients from the CNF Intake of free sugar, naturally occurring sugar and added sugars Canadian food group servings (2007) Canadian 2007 Healthy Eating Index score Healthy Eating Food Index (HEFI-2019) variable	Quantity of food and drink consumed Intake of 65 nutrients from CNF US Food Patterns Equivalents Canadian food group servings (2007) US Healthy Eating Index score

**Table 2:** Sociodemographic characteristics of participants (n = 68) and dropouts (n = 22).

Characteristics	Participants	Dropouts	*p* Values
	%		
Sex			
Female	71	60	0.37
Male	29	40	
Age in years			
18–30	43	45	0.50
31–50	43	50	
≥51	15	5	
BMI in kg/m^2^			
Normal weight, <25	63	50	0.38
Overweight, 25.0–29.9	23	22	
Obese, ≥30	14	27	
Education			
Trade school, high school, or no diploma	7	5	0.78
CEGEP	37	45	
University	56	50	
Annual gross household income in $ CAD			
<30,000	15	35	0.24
30,000–59,999	26	25	
60,000–99,999	25	15	
100,000 and more	33	25	
Health literacy categories			
High likelihood of low health literacy (score 0–1)	0	5	0.04
Possible low health literacy (score 2–3)	0	5	
Adequate health literacy (score 4–6)	100	90	

BMI: Body mass index; CEGEP: Collège d’enseignement général et professionnel; CAD: Canadian dollars; *p* value based on Chi-square tests.

**Table 3:** Usability of the R24W and ASA24-Canada-2018.

	R24W	ASA24-Canada-2018	*p* Values
SUS score ^1^, points (/100)	81 ± 2	58 ± 2	<0.0001
Completion time ^2^, min	14 (9–19)	25 (16–34)	<0.0001
Favorite tool reported ^3^, %	84	16	<0.0001

SUS: System usability scale; ^1^ Values (least squares means ± SDs) and *p* values were obtained from the mixed models and are adjusted for age and sex; ^2^ Values are median (Q1–Q3). *p* value based on Wilcoxon test; ^3^ Values are frequency. P-value based on Chi-square test.

**Table 4:** Most and least appreciated features of the R24W and ASA24-Canada-2018 among the 68 study participants.

	R24W	ASA24-Canada-2018
Most appreciated features	Simplicity and ease of use (N = 32) Photos of the portion size (N = 16) Variety of foods (N = 16) Reminders about frequently forgotten foods and potential additions (N = 8) Quick completion (N = 8) Search by category (drill down menu) and search tool with suggestions (N = 6) Tutorial (N = 4)	Photos of the portion size (N = 15) Visual appearance (N = 13) Reminders about frequently forgotten foods and potential additions (N = 11) Simplicity and ease of use (N = 11) Variety of foods (N = 9) Possibility to create your own recipe or to add a food manually (N = 4)
Least appreciated features	Some foods are not present in the database (N = 13) No possibility to create your own recipe or to add a food manually (N = 8) Visual appearance (N = 8) Completion time too long (N = 3)	Too many steps, details and questions that complicate the whole thing (N = 19) Reporting portions after listing all foods (N = 14) Difficulty to find food with the search tool (N = 13) Some foods are not present in the database (N = 13) Completion time too long (N = 6)

**Table 5:** Mean dietary intakes measured by the R24W and ASA24-Canada-2018.

	R24W	ASA24-Canada-2018	Differences	*p* Values
Energy, kcal	2282 ± 87	2252 ± 87	30	0.69
Vegetables and fruits, servings ^1^	5.5 ± 0.4	6.1 ± 0.4	0.6	0.07
Grain products, servings ^1^	5.0 ± 0.3	5.2 ± 0.3	0.2	0.74
Milk and alternatives, servings ^1^	2.0 ± 0.1	1.9 ± 0.1	0.8	0.38
Meat and alternatives, servings ^1^	2.2 ± 0.2	2.1 ± 0.2	0.1	0.42
Total Fat, g	93 ± 5	94 ± 5	1	0.81
Saturated Fat, g	32 ± 2	31 ± 2	1	0.44
Carbohydrate, g	266 ± 13	262 ± 13	4	0.68
Protein, g	91 ± 3	86 ± 3	5	0.11
Fibers, g	23 ± 2	23 ± 2	0	0.52
Sodium, mg	3201 ± 152	3341 ± 152	140	0.41

Values (least squares means ± SDs) and *p* values were obtained from the mixed models and are adjusted for age and sex; ^1^ Servings size defined according to the 2007 Canada’s Food Guide.

## Data Availability

The data presented in this study are available upon request to the corresponding author.

## Supplementary Materials

The following supporting information can be downloaded at: https://www.mdpi.com/article/10.3390/nu14214543/s1, Table S1: R24W and ASA24-Canada-2018 Setup.
