# Supplementary material for: Comparing the Usability of the Web-Based 24-h Dietary Recall R24W and ASA24-Canada-2018 among French-Speaking Adults from Québec
Source: Nutrients. 2022 Oct 28;14(21):4543. doi: 10.3390/nu14214543 (PMC9653863; doi:10.3390/nu14214543)
Supplement: Supplementary file 1 [file nutrients-14-04543-s001.zip › nutrients-1958565-supplementary.pdf]

Supplementary Materials

Table S1. R24W and ASA24-Canada-2018 Setup.

Parameters	Setting
Tool	24-hour recall
Study Type	Scheduled
Intake Time Frame	From Midnight to Midnight the previous day
Number of Logins Allowed	Multiple session
Time Provided to Complete Recall	24 hours
Modules	• Location?<br>• Ate With?<br>• TV/Computer On
Number of Recalls per respondent	2
Recall Distribution	Randomly distribute recalls per respondent across all days of the week
Minimal number of days between recalls	1
Attempts Numbers	3
